# Cytochrome P450 3A gene family and medication in childhood nephrotic syndrome: An update

**DOI:** 10.1016/j.gmg.2024.100009

**Published:** 2024-11-23

**Authors:** Praveenkumar Kochuthakidiyel Suresh, Yogalakshmi Venkatachalapathy, Sudha Ekambaram, Sangeetha, Megha Manoj, MohanaPriya C.D

**Affiliations:** a Sri Ramachandra Institute of Higher Education and Research, Department of Human Genetics, Chennai, Tamil Nadu 600116, India; b Apollo Children’s Hospital, Department of Pediatric Nephrology, 600006, India; c Sri Ramachandra Institute of Higher Education and Research, Department of Paediatric Medicine, Chennai, Tamil Nadu 600116, India; d Sri Ramachandra Institute of Higher Education and Research, Department of Bioinformatics, Chennai, Tamil Nadu 600116, India

**Keywords:** Cytochrome P450, Nephrotic syndrome, Single nucleotide polymorphism

## Abstract

**Background:**

Nephrotic syndrome (NS) is a renal disease characterized by excessive proteinuria (greater than 3.5 g/dl per 24 h), which results in hypoalbuminemia and leads to hyperlipidemia, edema, and various complications. NS patients typically respond to standard steroid treatment (prednisolone) and are classified as having steroid-sensitive nephrotic syndrome (SSNS). However, patients who do not respond to steroid therapy after 4 weeks are referred to as having steroid-resistant nephrotic syndrome (SRNS). The unequal response to steroid treatment in nephrotic syndrome involved many factors, including genetic, medication, and kidney diseases. The *CYP3A* gene family is predominantly involved in the metabolism of medications used in the treatment of NS.

**Methodology:**

A systematic literature review was conducted from January 2014 to June 2024 using an extensive electronic search of data related to pediatric nephrotic syndrome and the CYP gene family, including associated polymorphisms. Through this review, we systematically analyze factors that affect the metabolism of medications targeting the *CYP3A* gene family (including steroidal and non-steroidal drugs) commonly used in the treatment of NS and its comorbidities.

**Conclusion:**

Studies have correlated the relationship between polymorphisms in the *CYP3A* gene family and medication in NS, with 90 % of the research focusing primarily on post-kidney transplant NS patients. Many studies have reported a correlation between CYP3A gene family polymorphisms and increased tacrolimus (TAC) dosage.

## Introduction

Nephrotic syndrome (NS) is the second most common renal glomerular disorder in children, following congenital urinary tract and kidney abnormalities. The global prevalence of childhood NS is approximately 1 to 16 cases per 100,000 children [1]. Treatment commonly involves the use of corticosteroids and immunosuppressive agents. NS is categorized based on the response to corticosteroid therapy into steroid-resistant nephrotic syndrome (SRNS) and steroid-sensitive nephrotic syndrome (SSNS) [2]. Children of Asian descent are more likely to experience SSNS, while those of Latin American and African descent show a higher predisposition to SRNS. This suggests that genetic factors may influence both the prevalence of NS and the response to treatment [3]. In younger age groups, boys are more likely than girls to develop nephrotic syndrome [4]. Several biological factors may contribute to this phenomenon. One such factor is that women possess two X chromosomes, which contain immune-related genes. In contrast, men carry only one X chromosome. Additionally, the female sex hormone estrogen is believed to influence the immune system, suggesting its involvement in immunity-related processes [5]. Nephrotic syndrome can impact individuals across all age groups. Facial swelling is typically the initial symptom observed in most children affected by this condition. Conversely, adults commonly present with dependent edema, characterized by fluid accumulation in the lower extremities. It is commonly recognized that around 80 % of kids with nephrotic syndrome have a hereditary background of this condition, and the majority of them are therapy-resistant [6].

**Fig. 1:**
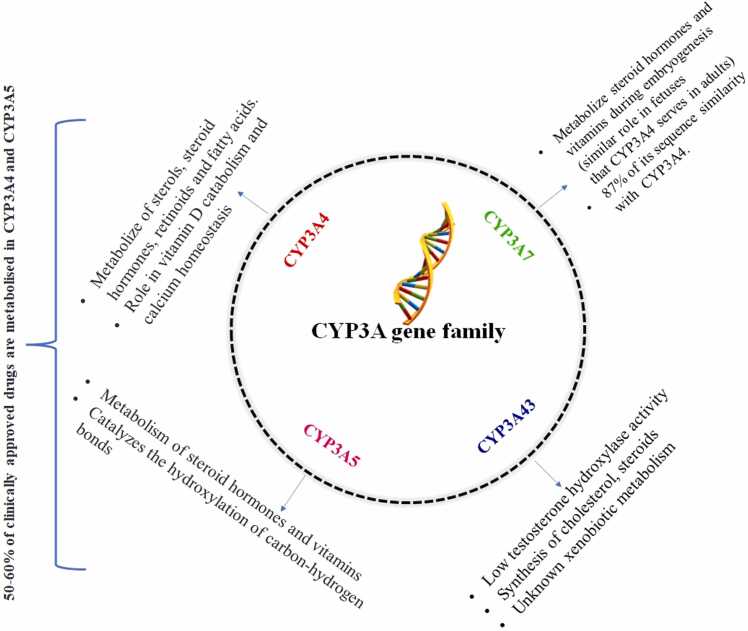
General function of cytochrome P450 gene family.

**Fig. 2:**
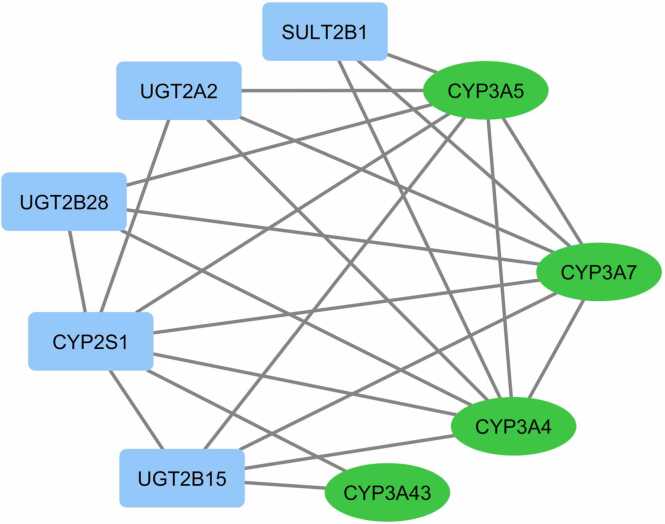
Genetic interaction associated with CYP3A gene family in nephrotic syndrome subjects (SRNS and SSNS) based on steroid response.

Glucocorticoids have served as the primary and widely used treatment for childhood nephrotic syndrome for many years. The administration of prednisone or prednisolone has been successful in achieving complete remission in the majority of affected children. Presently, there are established treatment protocols to manage the initial symptoms and relapses of nephrotic syndrome. Nevertheless, it's crucial to acknowledge that the disease's clinical course and the adverse effects of glucocorticoid therapy may differ significantly from person to person. Moreover, approximately 10 % of children diagnosed with nephrotic syndrome do not respond to steroid treatment and are categorized as steroid-resistant patients [7]. Here the significance of drug metabolism associated with Cytochrome P450, family 3, subfamily A, also known as CYP3A, gene group polymorphisms needs to get focused on. The human CYP3A enzyme family consists of four functional genes: *CYP3A4*, *CYP3A5*, *CYP3A7*, and *CYP3A43*. In this family *CYP3A4* and *CYP3A5*, the most prevalent enzymes metabolize more than half of all clinically administered medications **(**Fig. 1**)**. Hence the polymorphisms in the *CYP3A* gene family (*CYP3A4, CYP3A5*) have more importance in the assessment of drug metabolisms in NS; while *CYP3A7* is primarily involved in fetal drug metabolism and hemostasis, *CYP3A43* has a little functional or clinical significance. A recent study focused on pediatric patients to examine nephrotic range proteinuria and explored the connections between CYP3A5 * 3 gene polymorphism *rs776746* and *CYP3A7* gene polymorphisms *rs2257401* and *rs1021189 G* alleles, revealing that these correlations necessitate a higher tacrolimus dosage [8]. The clinical significance of prednisone/prednisolone-induced enzyme induction, on the other hand, remained unclear. However, the genotypic association of the *CYP3A* gene family in childhood nephrotic syndrome is less explored, which has major relevance concern in the assessment of drug metabolisms and consequent adverse drug reactions. *CYP3A* family genes interact with other gene groups and contribute to an effective metabolism **(**Fig. 2**)**. If the polymorphism or mutation in this effect affects any members, it affects the entire system and functionality, and it will also contribute to the pathogenesis of the disease. Fig. 2 represents the possible association of the *CYP3A* family with other groups of genes and pathways based on experimental and database similarity searches generated with the help of the String database [9] with a high confidence score.

## Method

**Fig. 3:**
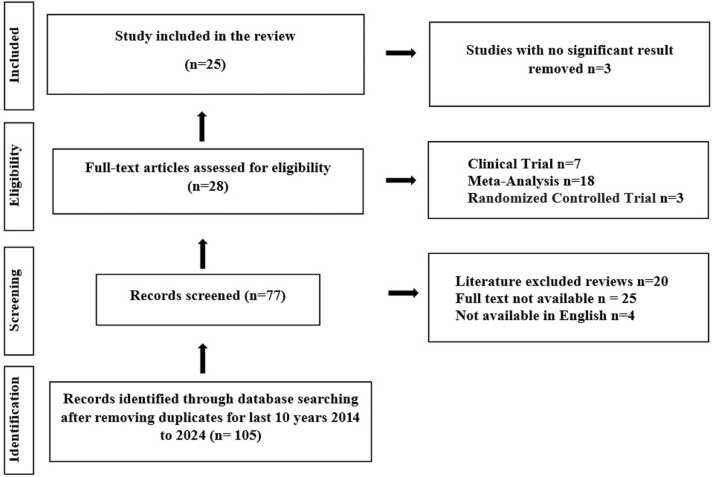
Literature screening flowchart.

A systematic literature review was conducted from January 2014 to June 2024, employing an extensive electronic search of data related to pediatric nephrotic syndrome and the CYP gene family. The search specifically targeted original and review articles, commentaries, editorials, letters, and case reports published in databases such as PubMed, Google Scholar, Scopus, and Elsevier. Manual removal of duplicates was performed, utilizing variables such as author, nationality, collaboration work, childhood nephrotic syndrome, and the CYP gene family. As a result, 25 relevant articles were extracted (Fig. 3). Additionally, we consulted current scientific literature and recommendations available on the Centre for Disease Control and Prevention (CDC) and World Health Organization (WHO) websites.

### Pathogenesis

Nephrotic syndrome is a renal disorder characterized by excessive protein excretion in the urine. It can affect individuals across all age groups; in adults, the prevalence of nephrotic syndrome differs compared to children in the ratio of 26:1 [10]. Diagnosing this condition in children can be challenging. The usual diagnostic criterion involves the excretion of 3.5 g/dl of total protein, out of 2.5 g/dl being albumin, within a 24-hour period [11]. Common symptoms include severe edema, particularly around the eyes, ankles, and feet, resulting in weight gain and foamy urine due to excessive protein in the urine.

### Risk factors for nephrotic syndrome

#### Medical conditions that can cause kidney damage

In addition, infections caused by HIV, hepatitis B, hepatitis C, and malaria have been identified as factors that increase the risk of developing nephrotic syndrome [12], [13].

### Medications and infection

Certain factors have been linked to the possibility of triggering nephrotic syndrome, which includes several anti-inflammatory and antibiotic drugs (ampicillin, rifampicin, and norfloxacin) [14]. In addition, infections caused by HIV, hepatitis B, hepatitis C, and malaria have been identified as factors that increase the risk of developing nephrotic syndrome [15].

### Pharmacological approaches for managing nephrotic syndrome

A wide range of treatment options are available to treat NS, ranging from steroids to calcium channel blockers (Table 3 a and b). The treatment option is purely based on the severity, ethnicity, region, comorbidities, etc.

**Table 1:** Major organization in nephrotic syndrome-associated study projects.

Database	Uses	URL	References
Nephrotic Syndrome Study Network (NEPTUNE)	Translational research infrastructure for Nephrotic Syndrome. This includes a longitudinal observational cohort study, a pilot and ancillary studies program, a training program, and a patient contact registry.	https://www.neptune-study.org/	[38]
EURenOmics	Develop novel tools for more accurate diagnoses and new and better therapies for rare kidney diseases including nephrotic syndrome	http://www.eurenomics.eu/the-project/wp02-steroid-resistant-nephrotic-syndrome/	[39]
CHILDNEPH	A national initiative to improve care and outcomes for patients with nephrotic syndrome	https://www.childneph.org/	[40]
PodoNet Registry	Explores the demographics, causes, and prognosis of patients with congenital and steroid-resistant nephrotic syndrome	http://www.podonet.org/index.php?id=home	[41]

**Table 2:** National library resources for nephrotic syndrome.

Agencies	Country	Year of Establish	URL
General catalogue	France	1461	https://catalogue.bnf.fr/ark:/12148/cb12520979g
Data	Italy	2011	https://data.bnf.fr/en/12520979/syndrome_nephrotique/
National Library of Israel	Israel	1892	http://uli.nli.org.il/F/?func=find-b&local_base=NLX10&find_code=UID&request= 987007565632005171
The Library of Congress	United States	1800	https://id.loc.gov/authorities/subjects/sh85090863.html
Web NDL Authorities	Japan	1997	https://id.ndl.go.jp/auth/ndlsh/00568067
Databases of the National Library CR	Czech Republic	1348	https://aleph.nkp.cz/F/?func=find-c&local_base=aut&ccl_term=ica=ph1141786&CON_LNG=ENG

**Table 3:** a & b Medication and its target gene.

	**Drug name**	**Drugbank id**	**PubChem compound id**	**Targets**	**Uniprot id**	**Uniprot Name**	**General function**	**PMID:**
Immune system-suppressing medications	Levamisole	DB00848	446541	*CHRNA3*	P32297	Neuronal acetylcholine receptor subunit alpha−3	Ligand-gated ion channel activity	17139284
				*ALPPL2*	P10696	Alkaline phosphatase, placental-like	Metal ion binding	10951365
				*unc−38*	Q23022	Acetylcholine receptor subunit alpha-type unc−38	Acetylcholine-activated cation-selective channel activity	9221782
				*unc−63*	Q9N587	Acetylcholine receptor subunit alpha-type unc−63	Acetylcholine-gated cation channel activity	15280391
				*lev−1*	Q27218	Acetylcholine receptor subunit beta-type lev−1	Acetylcholine-activated cation-selective channel activity	15990870
				*unc−29*	P48181	Acetylcholine receptor subunit beta-type unc−29	Acetylcholine-activated cation-selective channel activity	15990870
	Cyclophosphamide	DB00531		*NR1I2*	O75469	Nuclear receptor subfamily 1 group I member 2	Zinc ion binding	11297522
	Cyclosporine	DB00091	5284373	*CAMLG*	P49069	Calcium signal-modulating cyclophilin ligand	Likely involved in the mobilization of calcium as a result of the TCR/CD3 complex interaction. Binds to cyclophilin B.	23041287
				*PPP3R2*	Q96LZ3	Calcineurin subunit B type 2	Calcium ion binding	8524402
				*PPIA*	P62937	Peptidyl-prolyl cis-trans isomerase A	Virion binding	2001362
				*PPIF*	P30405	Peptidyl-prolyl cis-trans isomerase F, mitochondrial	Peptidyl-prolyl cis-trans isomerase activity	20676357
	Tacrolimus	DB00864	445643	*FKBP1A*	P62942	Peptidyl-prolyl cis-trans isomerase FKBP1A	Type I transforming growth factor beta receptor binding	16720724
	Mycophenolate	DB01024	446541	*IMPDH2*	P12268	Inosine−5′-monophosphate dehydrogenase 2	Rna binding	7903306
				*IMPDH1*	P20839	Inosine−5′-monophosphate dehydrogenase 1	Rna binding	15570183
	Rituximab	DB00073	46505820	*MS4A1*	P11836	B-lymphocyte antigen CD20	Mhc class ii protein complex binding	3925015
	Prednisolone	DB00860	5755	*NR3C1*	P04150	Glucocorticoid receptor	Zinc ion binding	27120390
Blood pressure medications	Lisinopril	DB00722	5362119	*ACE*	P12821	Angiotensin-converting enzyme	Zinc ion binding	17618628
				*REN*	P00797	Renin	Receptor binding	25767283
	Benazepril	DB00542	5362124	*ACE*	P12821	Angiotensin-converting enzyme	Zinc ion binding	17618628
	Captopril	DB01197	44093	*ACE*	P12821	Angiotensin-converting enzyme	Zinc ion binding	17618628
				*MMP2*	P08253	72 kDa type IV collagenase	Zinc ion binding	17308006
				*MMP9*	P14780	Matrix metalloproteinase−9	Zinc ion binding	2551898
				*LTA4H*	P09960	Leukotriene A−4 hydrolase	Zinc ion binding	11917124
				*BDKRB1*	P46663	B1 bradykinin receptor	Peptide binding	11880373
	Enalapril	DB00584	5388962	*ACE*	P12821	Angiotensin-converting enzyme	Zinc ion binding	17618628
	Losartan	DB00678	3961	*AGTR1*	P30556	Type−1 angiotensin II receptor	Protein heterodimerization activity	11752352
	Valsartan	DB00177	60846	*AGTR1*	P30556	Type−1 angiotensin II receptor	Protein heterodimerization activity	11752352
Diuretics	Furosemide	DB00695	3440	*SLC12A1*	Q13621	Solute carrier family 12 member 1	Sodium:potassium:chloride symporter activity	10653443
				*CA2*	P00918	Carbonic anhydrase 2	Zinc ion binding	1909891
				*GPR35*	Q9HC97	G-protein coupled receptor 35	G-protein coupled receptor activity	22236570
	Spironolactone	DB00421	5833	*NR3C2*	P08235	Mineralocorticoid receptor	Zinc ion binding	11791081
				*AR*	P10275	Androgen receptor	Zinc ion binding	18819053
				*PGR*	P06401	Progesterone receptor	Zinc ion binding	20650892
				*NR3C1*	P04150	Glucocorticoid receptor	Zinc ion binding	27120390
				*CYP11B2*	P19099	Cytochrome P450 11B2, mitochondrial	Steroid 11-beta-monooxygenase activity	976190
				*CYP17A1*	P05093	Steroid 17-alpha-hydroxylase/17,20 lyase	Steroid 17-alpha-monooxygenase activity	9452426
				*SHBG*	P04278	Sex hormone-binding globulin	Androgen binding	17881754
				*NR1I2*	O75469	Nuclear receptor subfamily 1 group I member 2	Zinc ion binding	16054614
	Hydrochlorothiazide	DB00999	3639	*SLC12A3*	P55017	Solute carrier family 12 member 3	Transporter activity	21613606
				*KCNMA1*	Q12791	Calcium-activated potassium channel subunit alpha−1	Voltage-gated potassium channel activity	29330545
	Metolazone	DB00524	4170	*SLC12A3*	P55017	Solute carrier family 12 member 3	Transporter activity	21613606
Cholesterol-reducing medications	Atorvastatin	DB01076	60823	*HMGCR*	P04035	3-hydroxy−3-methylglutaryl-coenzyme A reductase	NADPH binding	2991281
				*DPP4*	P27487	Dipeptidyl peptidase 4	Virus receptor activity	10951221
				*AHR*	P35869	Aryl hydrocarbon receptor	Transcription regulatory region DNA binding	30373764
				*HDAC2*	Q92769	Histone deacetylase 2	Transcription factor binding	28497810
				*NR1I3*	Q14994	Nuclear receptor subfamily 1 group I member 3	Zinc ion binding	28536098
	Fluvastatin	DB01095	1548972	*HMGCR*	P04035	3-hydroxy−3-methylglutaryl-coenzyme A reductase	NADPH binding	2991281
				*HDAC2*	Q92769	Histone deacetylase 2	Transcription factor binding	28497810
	Lovastatin	DB00227	53232	*HMGCR*	P04035	3-hydroxy−3-methylglutaryl-coenzyme A reductase	NADPH binding	2991281
				*ITGAL*	P20701	Integrin alpha-L	Metal ion binding	11812992
				*HDAC2*	Q92769	Histone deacetylase 2	Transcription factor binding	18381445
	Rosuvastatin	DB01098	446157	*HMGCR*	P04035	3-hydroxy−3-methylglutaryl-coenzyme A reductase	NADPH binding	2991281
				*ITGAL*	P20701	Integrin alpha-L	Metal ion binding	11812992
	Pravastatin	DB00175	54687	*HMGCR*	P04035	3-hydroxy−3-methylglutaryl-coenzyme A reductase	NADPH binding	2991281
				*HDAC2*	Q92769	Histone deacetylase 2	Transcription factor binding	28497810
	Simvastatin	DB00641	54454	*HMGCR*	P04035	3-hydroxy−3-methylglutaryl-coenzyme A reductase	NADPH binding	2991281
				*ITGAL*	P20701	Integrin alpha-L	Metal ion binding	11812992
				*HDAC2*	Q92769	Histone deacetylase 2	Transcription factor binding	28497810
Anticoagulants	Heparin	heparin	**46507594**	*SERPINC1*	P01008	Antithrombin-III	Serine-type endopeptidase inhibitor activity	17537059
				*F10*	P00742	Coagulation factor X	Serine-type endopeptidase activity	17118432
				*SELP*	P16109	P-selectin	Sialic acid binding	17458940
				*FGFR4*	P22455	Fibroblast growth factor receptor 4	Protein tyrosine kinase activity	11278860
				*FGF4*	P08620	Fibroblast growth factor 4	Heparin-binding	11486033
				*FGF19*	O95750	Fibroblast growth factor 19	Fibroblast growth factor receptor binding	14730967
				*FGFR1*	P11362	Fibroblast growth factor receptor 1	Protein tyrosine kinase activity	15096041
				*FGF1*	P05230	Fibroblast growth factor 1	S100 protein binding	16219767
				*FGFR2*	P21802	Fibroblast growth factor receptor 2	Protein tyrosine kinase activity	12529371
				*FGF2*	P09038	Fibroblast growth factor 2	Ligand-dependent nuclear receptor transcription coactivator activity	17524524
				*PF4*	P02776	Platelet factor 4	Heparin-binding	20162249
				*HGF*	P14210	Hepatocyte growth factor	Serine-type endopeptidase activity	15167892
	Warfarin	DB00682	54678486	*VKORC1*	Q9BQB6	Vitamin K epoxide reductase complex subunit 1	Vitamin-k-epoxide reductase (warfarin-sensitive) activity	14765194
				*NR1I2*	O75469	Nuclear receptor subfamily 1 group I member 2	Zinc ion binding	11297522
	Apixaban	DB06605	10182969	*F10*	P00742	Coagulation factor X	Serine-type endopeptidase activity	17371192
	Rivaroxaban	DB06228	9875401	*F10*	P00742	Coagulation factor X	Serine-type endopeptidase activity	17118432

Common name	Drugbank id	Genes Abbreviation	Uniprot id	Uniport Gene name	General function	P
Cyclophosphamide	DB00531	*CYP2C9*	P11712	Cytochrome P450 2C9	Steroid hydroxylase activity	9435160
		*CYP3A4*	P08684	Cytochrome P450 3A4	Vitamin d3 25-hydroxylase activity	11555828
		*CYP2B6*	P20813	Cytochrome P450 2B6	Steroid hydroxylase activity	21289075
		*CYP2C19*	P33261	Cytochrome P450 2C19	Steroid hydroxylase activity	18577768
		*CYP2A6*	P11509	Cytochrome P450 2A6	Steroid hydroxylase activity	11695850
		*CYP2C18*	P33260	Cytochrome P450 2C18	Steroid hydroxylase activity	11093772
		*CYP3A5*	P20815	Cytochrome P450 3A5	Oxygen binding	2732228
		*CYP2C8*	P10632	Cytochrome P450 2C8	Steroid hydroxylase activity	7574697
Cyclosporine	DB00091	*CYP2C19*	P33261	Cytochrome P450 2C19	Steroid hydroxylase activity	18577768
		*CYP2D6*	P10635	Cytochrome P450 2D6	Steroid hydroxylase activity	18698000
		*CYP3A5*	P20815	Cytochrome P450 3A5	Oxygen binding	2732228
Tacrolimus	DB00864	*CYP3A4*	P08684	Cytochrome P450 3A4	Vitamin d3 25-hydroxylase activity	10681376
		*CYP3A5*	P20815	Cytochrome P450 3A5	Oxygen binding	2732228
Mycophenolate	DB01024	*UGT2B7*	P16662	UDP-glucuronosyltransferase 2B7	Glucuronosyltransferase activity	10702251
		*UGT1A8*	Q9HAW9	UDP-glucuronosyltransferase 1 −8	Steroid binding	15472229
		*UGT1A9*	O60656	UDP-glucuronosyltransferase 1 −9	Retinoic acid binding	12181437
		*UGT1A1*	P22309	UDP-glucuronosyltransferase 1 −1	Steroid binding	12181437
		*UGT1A7*	Q9HAW7	UDP-glucuronosyltransferase 1 −7	Retinoic acid binding	12181437
		*UGT1A10*	Q9HAW8	UDP-glucuronosyltransferase 1 −10	Protein kinase c binding	15258099
		*CYP3A4*	P08684	Cytochrome P450 3A4	Vitamin d3 25-hydroxylase activity	10681376
		*CYP3A5*	P20815	Cytochrome P450 3A5	Oxygen binding	2732228
		*CYP2C8*	CYP2C8	Cytochrome P450 2C8	Steroid hydroxylase activity	7574697
Prednisolone	DB00860	*CYP3A4*	P08684	Cytochrome P450 3A4	Vitamin d3 25-hydroxylase activity	10681376
Benazepril	DB00542	*MTHFR*	P42898	Methylenetetrahydrofolate reductase	Protein complex binding	29891918
Losartan	DB00678	*CYP2C9*	P11712	Cytochrome P450 2C9	Steroid hydroxylase activity	7574697
		*CYP3A4*	P08684	Cytochrome P450 3A4	Vitamin d3 25-hydroxylase activity	10681376
		*CYP2C19*	P33261	Cytochrome P450 2C19	Steroid hydroxylase activity	18577768
		*UGT1A1*	P22309	UDP-glucuronosyltransferase 1 −1	Steroid binding	12181437
		*UGT1A3*	P35503	UDP-glucuronosyltransferase 1 −3	Retinoic acid binding	15472229
		*UGT1A10*	Q9HAW8	UDP-glucuronosyltransferase 1 −10	Protein kinase c binding	15258099
		*UGT2B7*	P16662	UDP-glucuronosyltransferase 2B7	Glucuronosyltransferase activity	10702251
		*UGT2B17*	O75795	UDP-glucuronosyltransferase 2B17	Glucuronosyltransferase activity	8798464
		*CYP2C8*	P10632	Cytochrome P450 2C8	Steroid hydroxylase activity	7574697
		*CYP2C9*	P11712	Cytochrome P450 2C9	Steroid hydroxylase activity	9435160
Furosemide	DB00695	*PGD*	P52209	6-phosphogluconate dehydrogenase, decarboxylating	Phosphogluconate dehydrogenase (decarboxylating) activity	20235752
		*UGT1A1*	P22309	UDP-glucuronosyltransferase 1 −1	Steroid binding	12181437
Spironolactone	DB00421	*CYP11B1*	P15538	Cytochrome P450 11B1, mitochondrial	Steroid 11-beta-monooxygenase activity	18215163
		*CYP2C8*	P10632	Cytochrome P450 2C8	Steroid hydroxylase activity	7574697
Atorvastatin	DB01076	*CYP3A4*	P08684	Cytochrome P450 3A4	Vitamin d3 25-hydroxylase activity	11555828
		*CYP3A7*	P24462	Cytochrome P450 3A7	Oxygen binding	9555064
		*CYP2C8*	P10632	Cytochrome P450 2C8	Steroid hydroxylase activity	7574697
		*CYP2D6*	P10635	Cytochrome P450 2D6	Steroid hydroxylase activity	18698000
		*CYP2C9*	P11712	Cytochrome P450 2C9	Steroid hydroxylase activity	9435160
		*CYP2C19*	P33261	Cytochrome P450 2C19	Steroid hydroxylase activity	18577768
		*CYP2B6*	P20813	Cytochrome P450 2B6	Steroid hydroxylase activity	21289075
		*UGT1A1*	P22309	UDP-glucuronosyltransferase 1 −1	Steroid binding	12181437
		*UGT1A3*	P35503	UDP-glucuronosyltransferase 1 −3	Retinoic acid binding	15472229
Fluvastatin	DB01095	*CYP1A1*	P04798	Cytochrome P450 1A1	Vitamin d 24-hydroxylase activity	11555828
		*CYP3A4*	P08684	Cytochrome P450 3A4	Vitamin d3 25-hydroxylase activity	11555828
		*CYP3A5*	P20815	Cytochrome P450 3A5	Oxygen binding	2732228
		*CYP2C8*	P10632	Cytochrome P450 2C8	Steroid hydroxylase activity	7574697
		*CYP2C9*	P11712	Cytochrome P450 2C9	Steroid hydroxylase activity	9435160
		*CYP2C19*	P33261	Cytochrome P450 2C19	Steroid hydroxylase activity	18577768
		*CYP2D6*	P10635	Cytochrome P450 2D6	Steroid hydroxylase activity	18698000
		*UGT1A1*	P22309	UDP-glucuronosyltransferase 1 −1	Steroid binding	12181437
		*UGT1A3*	P35503	UDP-glucuronosyltransferase 1 −3	Retinoic acid binding	15472229
		*UGT2B7*	P16662	UDP-glucuronosyltransferase 2B7	Glucuronosyltransferase activity	10702251
		*CYP2B6*	P20813	Cytochrome P450 2B6	Steroid hydroxylase activity	21289075
Lovastatin	DB00227	*CYP3A4*	P08684	Cytochrome P450 3A4	Vitamin d3 25-hydroxylase activity	11555828
		*PON3*	Q15166	Serum paraoxonase/lactonase 3	Protein homodimerization activity	10931838
		*CYP2C8*	P10632	Cytochrome P450 2C8	Steroid hydroxylase activity	7574697
		*UGT1A1*	P22309	UDP-glucuronosyltransferase 1 −1	Steroid binding	12181437
		*UGT1A3*	P35503	UDP-glucuronosyltransferase 1 −3	Retinoic acid binding	15472229
		*UGT2B7*	P16662	UDP-glucuronosyltransferase 2B7	Glucuronosyltransferase activity	10702251
		*CYP2C19*	P33261	Cytochrome P450 2C19	Steroid hydroxylase activity	18577768
Rosuvastatin	DB01098	*CYP2C9*	P11712	Cytochrome P450 2C9	Steroid hydroxylase activity	9435160
Simvastatin	DB00641	*CYP3A4*	P08684	Cytochrome P450 3A4	Vitamin d3 25-hydroxylase activity	11555828
		*CYP3A5*	P20815	Cytochrome P450 3A5	Oxygen binding	2732228
		*CYP2C8*	P10632	Cytochrome P450 2C8	Steroid hydroxylase activity	7574697
		*CYP2C9*	P11712	Cytochrome P450 2C9	Steroid hydroxylase activity	9435160
		*CYP2D6*	P10635	Cytochrome P450 2D6	Steroid hydroxylase activity	18698000
		*CYP2B6*	P20813	Cytochrome P450 2B6	Steroid hydroxylase activity	21289075
		*UGT1A1*	P22309	UDP-glucuronosyltransferase 1 −1	Steroid binding	27757045
		*UGT1A3*	P35503	UDP-glucuronosyltransferase 1 −3	Retinoic acid binding	15472229
		*UGT2B7*	P16662	UDP-glucuronosyltransferase 2B7	Glucuronosyltransferase activity	10702251
		*CYP2C19*	P33261	Cytochrome P450 2C19	Steroid hydroxylase activity	18577768
		*CES2*	O00748	Cocaine esterase	Methylumbelliferyl-acetate deacetylase activity	33424602
		*CES1*	P23141	Liver carboxylesterase 1	Triglyceride lipase activity	7980644
Heparin	DB01109	*HPSE*	Q9Y251	Heparanase	Syndecan binding	12213822
Warfarin	DB00682	*CYP2C9*	P11712	Cytochrome P450 2C9	Steroid hydroxylase activity	9435160
		*CYP1A2*	P05177	Cytochrome P450 1A2	Oxidoreductase activity, acting on paired donors, with incorporation or reduction of molecular oxygen, reduced flavin or flavoprotein as one donor, and incorporation of one atom of oxygen	19515014
		*CYP2C19*	P33261	Cytochrome P450 2C19	Steroid hydroxylase activity	18577768
		*CYP3A4*	P08684	Cytochrome P450 3A4	Vitamin d3 25-hydroxylase activity	11555828
		*CYP2C8*	P10632	Cytochrome P450 2C8	Steroid hydroxylase activity	7574697
		*CYP2C18*	P33260	Cytochrome P450 2C18	Steroid hydroxylase activity	11093772
Apixaban	DB06605	*CYP3A4*	P08684	Cytochrome P450 3A4	Vitamin d3 25-hydroxylase activity	11555828
		*CYP1A2*	P05177	Cytochrome P450 1A2	Oxidoreductase activity, acting on paired donors, with incorporation or reduction of molecular oxygen, reduced flavin or flavoprotein as one donor, and incorporation of one atom of oxygen	19515014
		*CYP2C8*	P10632	Cytochrome P450 2C8	Steroid hydroxylase activity	7574697
		*CYP2C9*	P11712	Cytochrome P450 2C9	Steroid hydroxylase activity	9435160
		*CYP2C19*	P33261	Cytochrome P450 2C19	Steroid hydroxylase activity	18577768
		*CYP2J2*	P51589	Cytochrome P450 2J2	Steroid hydroxylase activity	8631948
		*CYP3A5*	P20815	Cytochrome P450 3A5	Oxygen binding	2732228
Rivaroxaban	DB06228	*CYP2J2*	P51589	Cytochrome P450 2J2	Steroid hydroxylase activity	8631948
		*CYP3A5*	P20815	Cytochrome P450 3A5	Oxygen binding	2732228
		*CYP3A4*	P08684	Cytochrome P450 3A4	Vitamin d3 25-hydroxylase activity	11555828

Medication and enzymes

The response to steroid therapy in nephrotic syndrome (NS) varies among individuals. Studies have extensively documented the involvement of *CYP3A* gene polymorphism in post-kidney transplanted NS patients receiving steroids and immunosuppressant drugs. Genetic factors have been implicated in the resistance to steroid therapy. Consequently, several patients have been subjected to the identification of various genetic alterations in the *CYP3A* gene family, revealing differences in treatment response. Similarly, individuals with similar *CYP3A* gene mutations and polymorphism findings have displayed diverse responses to therapy, with some showing a positive response while others did not [16].

### Immunosuppressant drug association

Most research studies indicate an association between *CYP3A* gene polymorphism and drug dosage in NS individuals who have undergone kidney transplantation. Specifically, this relationship is prominent concerning the immunosuppressant drug Tacrolimus, which shows significant effects on its pharmacokinetics and clinical outcomes in solid organ transplant recipients due to *CYP3A5* gene polymorphism [17]. In pediatric subjects with nephrotic range proteinuria, Liu H *et al.* investigated the correlation between tacrolimus concentration and the genetic variants of CYP3A5 * 3 rs776746 and *CYP3A7 rs2257401, rs10211*. The study identified that a significant increase in tacrolimus dosages might be necessary for individuals with CYP3A5 * 3 A, CYP3A7 rs2257401 C, and *CYP3A7 rs10211 G* alleles, and it suggested the children’s with this polymorphism be used as a genetic marker to predict tacrolimus concentration and choose the appropriate dose [8], [18], [47].

The responses of patients with idiopathic nephrotic syndrome (INS) to prednisolone were evaluated based on the polymorphism *rs776746* in the *CYP3A5* gene. Depending on the types of medications employed in the treatment, the *CYP3A5 A6986G* polymorphism's impact may vary. A Taiwanese study suggested that in patients with nephrotic syndrome, the *rs776746* polymorphism of the *CYP3A5* gene showed a tendency towards association but did not reach statistical significance [19]. As the influence of *CYP3A5* gene polymorphisms increases, the response to prednisolone in children with idiopathic nephrotic syndrome (INS) becomes intricate. Moreover, investigations regarding the steroid response in children with INS might center around the steroid and xenobiotic receptor (SXR), which gets activated by various xenobiotics to regulate the expression of the *CYP3A4* gene [20].

In contrast to the above studies, it has been reported that CYP3A genetic factors (*rs776746, rs4646437, rs2257401, and rs10211*) exhibit variations at different stages post-transplantation. In a large cohort of patients with nephrotic syndrome (NS) treated with oral cyclophosphamide, more than half of the children experienced a relapse within the first 12 months following the cyclophosphamide treatment. Only 20 % of the children achieved a prolonged remission. [21], [22].

### Calcium channel blocker

Diltiazem, a calcium channel blocker, is typically prescribed to manage blood pressure in nephrotic syndrome patients, often in combination with tacrolimus (TAC). A lower TAC weight-normalized dose was necessary with the addition of diltiazem. It's important to note that CYP3A5 * 3 * 3 carriers showed this behavior more than other carriers. The primary metabolic enzyme of TAC is CYP3A5, whose affinity was comparable to the CYP3A4 gene while having a catalytic efficiency that was 64 % greater. The concurrent level of CYP3A4 has a significant impact on the relative contribution of CYP3A5. When *CYP3A5* loses its expression and functionality, TAC metabolism is then undertaken by *CYP3A4*. The use of diltiazem in combination with this could therefore reduce the activity of CYP3A4 when CYP3A5 is impaired, leading to a considerable decrease in TAC metabolism. The recommended TAC dosage of 0.5 mg per capsule poses a challenge in accurately providing the required daily dose when it doesn't align with an integer multiple of 0.5. However, it is crucial to take into account the possibility of regulating the daily dose of diltiazem, which becomes another important factor to consider while using the medication. Achieving a target concentration of 5 ng/ml of TAC for a 20 kg child with the CYP3A5 * 1/* 3 genotype requires a daily dose of approximately 1.7 mg. Nevertheless, in practical terms, administering this dosage can be challenging. The TAC concentration in children may be excessively high if 2 mg is given, which could result in negative effects such as nephrotoxicity. The recommended clinical advice for this child would be to take 1.5 mg of TAC along with 60 mg of diltiazem. This approach not only helps prevent excessive medication concentration but also offers potential benefits in terms of kidney protection [23].

### Anti-fungal medication

The simultaneous use of “azole” antifungal medications is notable due to the significant pharmacological interaction experienced by individuals with the CYP3A5 * 1 genetic variant. Rituximab treatment has demonstrated promise in managing steroid-dependent nephrotic syndrome. However, its efficacy in preventing relapses has proven to be predominantly temporary for the majority of patients [24].

### Biological agents and the CYP3A gene family

Biological agents constitute a significant class of drugs that possess both therapeutic efficacy and substantial importance, including the following:.

### Black pepper

Black pepper (Piper nigrum) is commonly used as a seasoning agent and has medicinal properties. However, outrageous consumption or intake through dietary supplements containing more than ten mg of piperine or piper amides may lead to clinically significant interactions, such as the inhibition of *CYP3A4* enzymes [42].

### Schisandra

Schizandra chinensis fruit is a vital component of various traditional Asian medicines, often used for their hepatoprotective properties. Current clinical data strongly indicate that Schisandra extracts pose a significant risk of increasing blood levels of medications that are CYP3A substrates. This effect is due to the inhibition of human CYP3A4 and CYP3A5 enzymes by gomisin C and gomisin G, two lignan analogs derived from Schisandra chinensis [43].

St John’s Wort.

St. John's Wort is used for its antidepressant properties, with hyperforin being the active substance and the most potent known activator of *PXR12*. Clinical studies have shown that products containing less than 1 % hyperforin are less likely to cause interactions. However, most products contain 3 % hyperforin, indicating that St. John's Wort is an inducer of *CYP3A4* [44].

### Grapefruit

Grapefruit (Citrus paradisi), in all its forms, is a potent inhibitor of intestinal *CYP3A4* and has been reported to interact with over 44 medications, potentially causing serious adverse effects. Healthcare professionals should inquire about patients' use of complementary and alternative medicines when prescribing medications metabolized by *CYP3A4*. The inhibitory potential on human *CYP3A* is ranked as follows: Citrus paradisi > Morus nigra > Vitis riparia > Punica granatum > Rubus occidentalis [45].

### Role of the CYP3A gene family in Nephrotic syndrome

The *CYP3A4* enzyme activity displays significant interindividual variability, with a range of up to 60-fold, leading to therapeutic failures, unexpected adverse effects, or severe drug toxicity. This wide variation in drug metabolism is primarily influenced by a combination of genetic polymorphisms, gene expression regulation, and interactions with drugs or environmental chemicals. Genetic polymorphisms contribute to approximately 90 % of the interindividual variability in *CYP3A4* activity, making them valuable in predicting an individual's response to certain drugs used in treatments. Additionally, *CYP3A4* polymorphisms have the potential to assist in predicting susceptibility to certain illnesses [25]. Among cytochrome P450 enzymes, *CYP3A* is highly prevalent in human livers, representing the most abundantly expressed subtype. It can account for up to 60 % of the total P450 content in certain samples. CYP3A enzymes play a crucial role in metabolizing various foreign substances (xenobiotics) and exhibit significant activity in the hydroxylation of steroids and bile acids. In adult human livers, four members of the CYP3A subfamily have been identified: *CYP3A3, CYP3A4, CYP3A5*, and *CYP3A43* [26]. Significant changes in *CYP3A* expressions and activities occur during fetal life, neonatal life, and the pediatric period, altering their catalytic function. The maturation patterns of *CYP3A7, CYP3A5*, and *CYP3A4* are age-dependent [22]. Hepatic metabolism, which includes phase I and phase II processes, is principally responsible for the elimination of prednisolone and prednisone from the body. The *CYP450* system is considered the primary enzyme system responsible for phase I metabolism. However, the degree of participation of certain cytochrome P450 (CYP)3A isoenzymes in prednisone/prednisolone metabolism has yet to be thoroughly elucidated. However, because of reduced clearance, co-administration of the potent *CYP3A4* inhibitor ketoconazole has been reported to increase both total and unbound prednisolone concentrations in plasma by approximately 50 %. The previous study has demonstrated that taking enzyme inducers together with prednisolone causes higher clearance and a shorter half-life [27]. The genetic factors that affect the pharmacokinetic or pharmacodynamic characteristics of patients can contribute to a wide range, from 20 % to 95 %, of the variability in the effectiveness and adverse effects of therapeutic agents. Genetic variations, or polymorphisms, in the *CYP3A5* gene have been found to explain approximately 40–50 % of the variation in tacrolimus dose requirements among individuals of Caucasian descent [28]. In a Chinese study involving juvenile patients with nephrotic range proteinuria, it was reported that non-carriers of the *rs2257401* C and *rs10211* G alleles had nearly twice the concentration of tacrolimus compared to carriers of these alleles. However, there is currently no published literature on the relationship between *CYP3A7 rs2257401* and *rs10211* and tacrolimus pharmacokinetics in children. It has been observed in Chinese adult patients at different transplantation stages that *CYP3A7 rs2257401* and *rs10211*can impact cyclosporine levels [8]. By activating *CYP3A4* and P-glycoprotein, glucocorticoids have been shown to alter the pharmacokinetics of other medications. The clinical significance of prednisone/prednisolone-induced enzyme induction, on the other hand, largely remained unclear; only a few studies are investigating the same.

## Discussion

Nephrotic syndrome (NS) is a chronic condition commonly affecting children, and the response to steroid therapy is generally successful, with success rates ranging from 64 % to 80 % with a reduced death rate of 3 % [29]. However, there are conflicting findings regarding the incidence of haematuria in girls compared to boys, with Mortazavi et al. reporting a higher incidence in girls and other studies showing the opposite [7]. The key characteristics of nephrotic syndrome include significant proteinuria, edema, hypoalbuminemia, and elevated levels of cholesterol [30]. In recent years, the incidence of childhood nephrotic syndrome has steadily increased [31]. Pulse therapy with glucocorticoids (GC) is considered an effective treatment approach, with prednisone achieving complete remission in most children. However, when high-dose intravenous methylprednisolone is unsuccessful, calcineurin inhibitors, such as cyclosporine and tacrolimus, are commonly used as initial treatments for such cases. The role of ethnicity has been identified as significant in the pathogenesis of nephrotic syndrome, as indicated by numerous studies. SSNS is prevalently seen in African and Latin American ethnicities, and SSNS is prevalent in Asian children [32]. The Cytochrome p450 3A family is mostly involved in the metabolism of corticosteroid and immunosuppressant medications [33]. The polymorphism involved in this gene family contributes to additional drug dosage and reduces the effectiveness of the drug group that is used in the treatment of the condition [34]. If the drug dosage is increased, it affects other systems in our body [35]. Steroid resistance patients are not responding properly to medication if the target of the drug of interest gets mutated or polymorphed [36]. Here the importance of target medication is the identification of potential drug target is important in the treatment of the condition hence the polymorphism identification in the target of medication needs to get more focus more studies are needed in this area. Genetic factors involved in drug metabolism could potentially contribute to steroid resistance in individuals with nephrotic syndrome (NS). Genetic factors involved in drug metabolism could potentially contribute to steroid resistance in individuals with nephrotic syndrome (NS) [37]. Popular dietary supplements and foods that have a high risk for interaction with medicines metabolized by the *CYP3A* gene family are mostly due to the members CYP3A4 and CYP3A5 [42], [43], [44], [45], [46]. Prevalently, most of the studies suggested that *CYP3A5* polymorphism contributes significantly lower the efficiency of tacrolimus dosage and forces patients to take the higher dosage in post-kidney transplant patients in NS [17]. The medication diltiazem co-administration with tacrolimus effectively reduces the tacrolimus dosage. The primary organization is actively involved in investigating the severity of nephrotic syndrome by funding projects aimed at identifying its impact on society. National library resources mentioned in Table 1, Table 2 provide valuable information and insights to enhance knowledge and understanding of these conditions. This collective effort is expected to contribute to improved management strategies for individuals affected by nephrotic syndrome.

## Conclusion

Treating individuals with nephrotic syndrome who are resistant to therapy or require prolonged treatment can be challenging, including steroid toxicity. Currently, no single performance-enhancing drug has been identified as superior for steroid-resistant nephrotic syndrome patients, and the drug selection often depends on geographical factors, including food. Ethnicity also plays a significant role in drug selection, dosage, clinical practice, and physician preferences, as suggested by numerous studies. The presence of *CYP3A* gene family polymorphism, which affects drug metabolism in nephrotic syndrome treatment, has emerged as an important factor for optimal drug dosage and improved management of the condition. Currently, three members of the *CYP3A* family are identified as involved in the polymorphism, namely *CYP3A4, CYP3A5*, and *CYP3A7*. The *CYP3A43* member does not contribute much to the polymorphism and metabolism of drugs used in the treatment of nephrotic syndrome.

## Ethics Statement

The authors are accountable for all aspects of the work in ensuring that questions related to the accuracy or integrity of any part of the work are appropriately investigated and resolved.

## Ethical approval

Not applicable.

## Funding

The authors received no financial support for the research, authorship and publication of this article.

## CRediT authorship contribution statement

All authors certify that they have participated in this work. Praveenkumar K S (Research scholar)- Conceptualization (Primary role), Data curation (Lead responsibility), Formal analysis (Lead involvement), Investigation (Lead responsibility). Yogalakshmi V (Research scholar)- **-** Writing – review & editing (Lead). Dr.Sudha Ekambaram (Senior Pediatric Nephrologist) Conceptualization (lead). Dr.G. Sangeetha (Assistant professor) - Conceptualization (Equal), Data curation (Equal), Formal analysis (Equal), Methodology (Equal). Megha Manoj (Graduate student) - Writing – review & editing (Equal). C.D. Mohana Priya (Associate professor) - Supervision (Primary role), Writing – review & editing (Equal contribution).

## Declaration of Competing Interest

The authors have no conflict of interest.

## Data Availability

All the data are provided in the manuscript any additional data required is available based on reasonable request from the corresponding author.
